# The validity of automatic methods for estimating skeletal age in young athletes: a comparison of the BAUSport ultrasound system and BoneXpert with the radiographic method of Fels

**DOI:** 10.5114/biolsport.2024.127380

**Published:** 2023-05-30

**Authors:** Sean P. Cumming, Ramon Pi-Rusiñol, Gil Rodas, Franchek Drobnic, Alan D. Rogol

**Affiliations:** 1 University of Bath, Bath, United Kingdom; 2 MD, Futbol Club Barcelona Medical Services, Barcelona, Spain; 3 FC Barcelona, Futbol Club Barcelona Medical Services, Barcelona, Spain; 4 School of Medicine, University of Virginia, Charlottesville, VA USA

**Keywords:** Puberty Assessment, Sport, Maturity, Ultrasound, X-ray

## Abstract

This study examined the validity of two automated methods (BAUSport, BoneXpert software using Fels, Greulich-Pyle, Tanner-Whithouse III protocols) for estimating skeletal age (SA) in young athletes in comparison to a reference standard (Fels). 85 male and female athletes, nine to seventeen years of age, from multiple sports were assessed for SA as part of an annual medical and health screening programme. Intra-class correlations demonstrated high degrees of association between the automatic methods for estimating SA (BAUSport r = .98; BoneXpert r = .96–.99) and the discrepancy between SA and chronological age (SA-CA) (BAUSport r = .93; BoneXpert r = .88–.97), with the reference standard. Concordance analyses for the categorisation of participants as early, on-time and late maturing also demonstrated substantial levels of agreement for both methods (BAUSport Kappa = .71; BoneXpert Fels Kappa = .63) with the reference standard. Bland-Altman plots comparing the automatic methods with the reference standard identified statistically significant fixed biases, ranging in magnitude from small to large. Collectively, these results suggest that BoneXpert and BAUSport can provide comparable estimates of SA and SA-CA in young athletes relative to the Fels method. Biases in the estimation of SA should, however, be considered and the automatic methods should be implemented as part of a comprehensive growth and maturity screening protocol. The non-invasive nature of the BAUSport method affords particular advantages (no radiation exposure, portability) in contexts where the regular estimation of SA is recommended.

## INTRODUCTION

The optimal development of young athletes requires a sound understanding and awareness of child development [1]. It is advised that sport’s national governing bodies implement practical and effective policies/procedures for assessing and monitoring growth and maturation in young athletes, and educate coaches, sports scientists, and medical practitioners on physical development in youth [1]. Individual differences in maturity status and timing impact athletic performance, athlete selection biases, training effects, and injury risk in young athletes. Information pertaining to the growth and maturation of young athletes can be used for several purposes. These include, (i) differentiating between athletes who are early, on-time, or delayed in maturation, (ii) more accurately evaluating physical fitness, athletic performance and future potential, (iii) identifying when athletes enter developmental stages where they may be at greater risk for injury (i.e., adolescent growth spurt), (iv) grouping athletes by maturity for training and/or competition (i.e., bio-banding), and/or (v) informing the design, implementation and evaluation of training and conditioning programmes [2, 3]. The effectiveness of these strategies is, however, dependent upon the validity and reliability of the methods used to estimate growth and/or maturation.

The processes of growth and maturation are related yet distinct [4]. Growth refers to changes in body size, composition, and/or physique; whereas maturation refers to the process of progress towards the adult or mature state [4]. Common measures of growth include height and weight, which can be assessed in terms of status (cm. or kg.) and/or velocity (e.g., gains in cm. or kg., per annum). Maturation occurs, and can be estimated, within multiple biological systems, including skeletal, dental, endocrine, sexual, and somatic characteristics.

Skeletal age (SA) is considered the most reliable and valid method for estimating maturation status and can be estimated from birth to late-adolescence [4]. Radiographs of the hand-wrist are generally used to estimate SA with several methods (protocols) available, including the Greulich-Pyle [5], Fels [6], and the Tanner-Whitehouse methods (TW1, TW2 & TW3) [7–9].

SA derived from radiographs of the hand-wrist provide valid and reliable estimates of biological maturation status in youth; however, this index is not without limitations [10]. Radiographs are expensive, time intensive, and require specialists trained in the use and interpretation of skeletal hand-wrist x-rays. Assessments of SA via x-ray also involves exposure to small radiation doses [11]. Although the dose presents minimal risk, decisions to request radiographs must provide evidence that the benefits of performing the procedure outweigh the potential health risks to the athlete. Consequently, the use of skeletal hand x-rays to estimate maturation status in young athletes is increasingly limited to cases where there are medical concerns regarding the growth/health/injury status of the child or when his/her chronological age is unknown.

Advances in digital imaging technologies and machine learning have led to the development of imaging software, such as BoneX-pert, that automatically estimates SA from digitalised skeletal hand-wrist radiographs [12]. BoneXpert uses a three-layer imaging process to (i) reconstruct and validate the bone borders and architecture (ii) determine and validate SA, and (iii) average and adjust SA to the Greulich-Pyle method and/or transform these values to the TW3 or Fels stages and estimates of SA. BoneXpert provides a standardised, cost effective, and less time-intensive alternative for estimating SA, yet still requires the procurement of the hand-wrist x-ray.

Ultrasound has been proposed as an alternative, automatic, and non-invasive method for estimating SA in youth [13]. Ultrasound methods estimate SA by deducing the velocity at which sound waves pass through specific bones sites, generally the distal radius and/or ulna epiphysis [13]. As ultrasound does not involve ionizing radiation, it presents no risk to the child and can be used more frequently. Strong correlations have been reported between estimates of SA derived from sonography and skeletal hand-wrist x-rays using the Greulich-Pyle method [13, 14]. These studies have, however, included broad age ranges from early childhood to late adolescence [13, 14]. Associations between estimates of biological maturation are inflated when considering children across broad age ranges and the capacity of sonographic methods to differentiate between children of varying maturity status within narrower age bands remains unclear. Existing sonographic methods have also been criticised for relying upon single sites of assessment and over-and under-estimating SA in late and early maturing youth, respectively [15].

A particular limitation of existing sonographic methods for estimating SA is the reliance upon single or limited numbers of bone sites (i.e., radius and/or ulna). The epiphyses of the radius and ulna are ideal sites as they are present from early childhood and represent two of the last bones in the hand-wrist to attain full maturity [4, 10].

Nevertheless, there is substantial variance in the rates and ages at which the radius and ulna achieve maturity [11], introducing the potential for significant error and limiting their suitability as *exclusive* sites for estimating SA. The validity and reliability of sonographic methods could be improved by increasing the number of sites within the assessment procedure. Emerging evidence suggests that sonographic techniques (BAUSport) that utilise multiple assessment sites (e.g., radius, ulna, carpals, phalanges) may provide more reliable and valid estimates of SA [16]. Further research examining the validity and reliability of these new methods is, however, warranted.

Considering the preceding discussion, the purpose of this investigation was to examine the validity of two automatic methods for estimating SA in a combination of male and female athletes. Specifically, estimates of SA and SA-CA derived from invasive (BoneXpert) and non-invasive (BAUSport) automatic methods for estimating SA were compared against estimates of SA derived from the Fels protocol. The capacity of both automatic methods to correctly identify participants as early, on-time and late maturing relative to the Fels protocol was also investigated. Bland Altman analyses were also performed to examine the degrees of agreement between the estimates of SA provided by the automatic methods and the Fels protocol. The Fels method was selected as the *reference standard*, as it uses a comprehensive and diverse set of criteria for estimating SA and includes an accompanying standard error [17].

## MATERIALS AND METHODS

### Participants

The sample include 85 male and (n = 13) female soccer, volleyball, handball, and basketball players registered with a multisport academy in Catalonia, Spain. Participants were aged between 9 and 17 years (M = 13.0 years, SD = 1.6 years). A posthoc power analysis for correlational analyses (G*Power version 3.1.9.6) [18] based upon current sample size, the lowest value for designating a large effect (r = 0.5), and a minimum probability value of .05, indicated sufficient statistical power (= .99). As all protocols for estimating skeletal age were sex specific, male and female participants were combined for all analyses. Further, there was not adequate statistical power to conduct the analyses for the female participants alone.

### Ethical procedures

Data collection was approved by Clinical Research Ethics Committee of the Sports Administration of Catalonia. Participants and their parents and/or guardians were informed of the nature and purpose of the study in advance of data collection before providing both written consent and assent for participation. Ethical approval for the analysis of anonymised data was approved by the Research Ethics Approval Committee for Health at the lead author’s host institution.

### Measures

The data collection was conducted over a 10-month period. Maturity status assessments were conducted following standardised procedures for skeletal hand-wrist x-rays and use of the BAUSport system. All participant assessments were conducted on a single day by the Academy’s Medical Service Department as part of the annual medical and health screening programme for registered athletes.

### Skeletal Age: Radiographs

Dorso-palmar radiographs of the left hand-wrist were procured to estimate skeletal age (SA) using the Fels method [6]. The x-ray examinations were performed using standardised procedures by two medical doctors, each with over 15 years’ experience in Paediatric Sports Medicine. Digital images (DICOM files) were then generated from each radiograph to estimate SA using the BoneXpert 3.0 imaging software [19]. The BoneXpert software provide estimates of SA in accordance with the Fels. Greulich-Pyle, and TW3 protocols. One participant’s DICOM image was unable to be processed by BoneXpert. Accordingly, this participant was excluded from all analyses pertaining that required estimates of SA derived from BoneXpert. Participants presenting an SA equal or greater to, or equal lesser than, one year of their chronological age were categorised as early or late maturing, respectively. Participants with a SA falling within +-1 years of their chronological age were categorised as ‘on time’.

SA was estimated independently by a single Academy medical doctor specialising in paediatric sports medicine who was trained in the use of the Fels protocol and associated software (Felshw.com) as part of his professional and medical training. A subsample of 20 of radiographs were also assessed by the lead author. Both assessors were blinded to one another’s SA estimates and the estimates derived from the BAUSport and BoneXpert systems. The intra-class correlation (ICCs) between the independent investigators’ estimates of SA using the Fels protocol was positive, strong, and statistically significant (r = .99, p < .001). The absolute (A.TEM) and relative technical errors of measurement (R.TEM) between the independent assessors estimates of SA using the Fels protocol across the subsample was .42 years and 3.2 percent, respectively, with the lead author reporting a slightly lower mean estimate for SA (-0.23 years).

### Skeletal Age: Ultrasound

The BAUSport instrument system with accompanying software, produced by SonicBone Medical Ltd., Rishon LeZion, Israel, was used to estimate SA based upon ultrasound assessment of three skeletal locations on the left hand-wrist. Assessments were conducted by three medical professionals in the academy’s Medical Services Department who were trained in the use of the BAUSport system. These sites include the distal radius and ulna’s secondary ossification centres on the epiphysis at the hand-wrist: the growth plate of metacarpal III and the shaft of the adjacent proximal phalange, and the distal metacarpal epiphysis. Information, based upon the speed at which high frequency waves of an ultrasound pulse propagate through bone and distance attenuation factors (i.e., decay rate), is fed into an integrated algorithm using the scoring method designed by Tanner and Whitehouse (TW2 method). The algorithm then provides the estimate of SA and future adult stature. The time durations for the scans at each of the various sites was 12 seconds for the radius and ulna, and four seconds for the proximal phalange and distal metacarpal. Total time for completing the assessment is approximately five-to-ten minutes per participant. The BAUSport system has previously demonstrated high levels of repeatability and validity in young athletes and the general population [16, 20–22].

### Statistical Analyses

A series of statistical analyses were conducted to the investigate the degree to which the automatic estimates of SA agreed with the reference standard (Fels), including ICCs to examine associations between the estimates of SA and SA-CA; A.TEM and R.TEM to determine the magnitude of the differences between the automatic estimates of SA with the reference standard; Bland-Altman plots to examine the degree to which the automatic methods estimates of SA agreed with the estimates provided by the reference method; one-sample mean T-tests to identify the presence of fixed effect biases between automatic estimates of SA and the Fels standard; and cross tabulation analyses using Cohen’s Kappa coefficient to determine the agreement amongst the methods in classifying participants as early, on-time, and late maturing.

### Outliers

Prior to the main analyses, the data were investigated for outliers. Outliers represent data points that differ significantly from other observations and may occur due to chance or experimental error. A strategy whereby any participant presenting an estimate of SA that differed by more than three years from at least two of the four SA estimates derived from other methods, was used to identify, and remove outliers. One skeletal year approximates one standard deviation in skeletal age among youth of the same age. Two male participants, approximating two percent of the original sample, were removed based upon SA estimates derived from the BAUSport (n = 1) and BoneXpert (n = 1) protocols and the exclusion criteria.

## RESULTS

### Descriptive analyses

Descriptive statistics for age, SA and the discrepancy between skeletal and chronological age (SA-CA) are presented for the total sample and by sex in Table 1. For all the automatic estimates of SA, except the BAUSport system, the reference method (Fels Practitioner) produced a higher mean value. Of note, all the mean values for SA in the male participants were higher than the equivalent value for chronological age; whereas the mean values for SA in the female participants approximated, or fell below, the mean value for chronological age.

**TABLE 1:** Descriptive statistics for chronological age and estimated skeletal age (SA) across methods by sex and for the total sample.

	Males (n = 70, ^a^69) M (SD)	Females (n = 13) M (SD)	Total (N = 83, ^c^82) M (SD)
Chronological age	13.3 (1.5)	11.5 (1.3)	13.0 (1.6)
SA FELS Practitioner	14.3 (2.3)	11.2 (1.6)	13.8 (2.4)
SA BAUSport	14.5 (2.4)	11.6 (1.8)	14.0 (2.5)
SA FELS BoneXpert	14.0 (2.3)^a^	11.0 (1.5)	13.5 (2.4)^c^
SA GP BoneXpert	13.8 (2.3)^a^	10.7 (1.5)	13.3 (2.5)^c^
SA TW3 BoneXpert	13.4 (2.2)^a^	10.3 (1.4)	12.9 (2.4)^d^

### Intra-class correlations

**FIG. 1:**
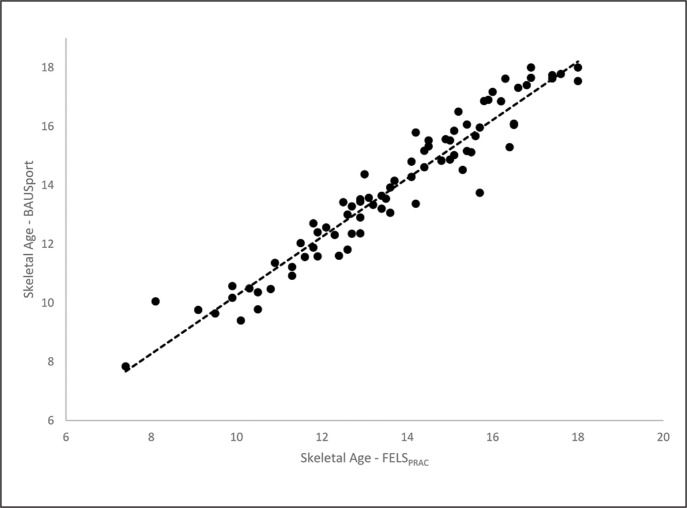
Intraclass correlations and scatterplots for estimates of skeletal age derived from the BAUSport system and Fels protocol.

**FIG. 2:**
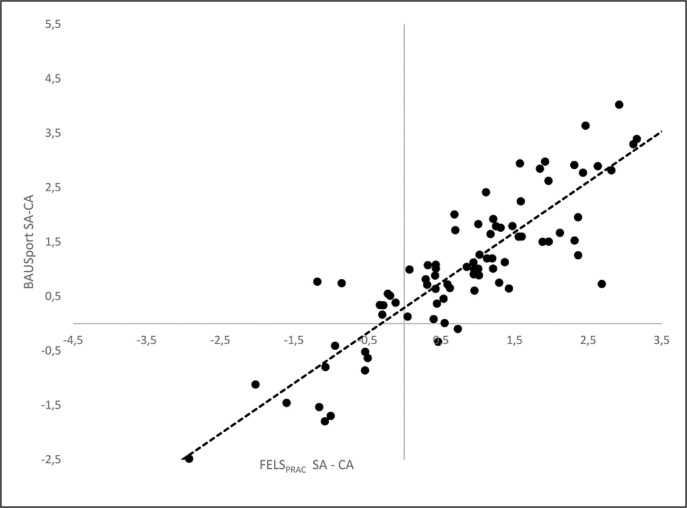
Intraclass correlations and scatterplots for estimates of skeletal age and the discrepancy between skeletal and chronological age (SA-CA) as estimated by the BAUSport systems and Fels protocol.

ICCs (one-tailed) using mixed effects and absolute agreement were performed to examine the magnitude and direction of the associations between the automatic estimates of SA and the reference method (Table 2). One-tailed analyses were selected on the basis that estimates of SA and SA-CA are expected to correlate positively across protocols. A separate series of equivalent analyses were conducted for the discrepancies between SA and chronological age (SA-CA) (Table 2). All estimates of SA and the SA-CA were positively and significantly correlated with the reference method. The correlations for SA were strong in magnitude ranging from .96 (BoneXpert TW3) to .99 (BoneXpert Fels). The correlations for SA-CA were also statistically significant and strong in magnitude yet presented a greater range of variation (BoneXpert TW3 r = .88; BoneXpert Fels r = .97). Accompanying scatterplots for the correlations between the non-in-vasive automatic method (BAUSport System) and the Fels method are presented in Figures 1 and 2 for SA and SA-CA, respectively.

**TABLE 2:** Comparison of methods for estimating skeletal age against the Fels method in male and female adolescent athletes aged 11 to 17 years.

	ICC SA	ICC SA-CA	A.TEM Years	R.TEM	Kappa
BAUSport	.98^c^	.93^c^	.49	3.49%	.71^c^
BoneXpert GP	.98^c^	.95^c^	.45	3.38%	.54^c^
BoneXpert TW3	.96^c^	.88^c^	.67	5.07%	.35^b^
BoneXpert Fels	.99^c^	.97^c^	.35	2.60%	.63^c^

Note: SA = skeletal age, CA = Chronological Age, ICC = Intraclass correlation, A.TEM = Absolute Technical Error of Measurement, R.TEM = Relative Technical Error of Measurement,

b = p < .01,

c = p < .001.

A.TEMs and R.TEMs were calculated for all estimates of SA, relative to the reference standard, and are presented in Table 2. The A.TEM. values ranged from .35 (BoneXpert Fels) to .67 (BoneXpert TW3) years. The R.TEM. values ranged from 2.60% (BoneXpert Fels) to 5.07% (BoneXpert TW3).

Cross tabulation analyses using percentage of agreement values and Cohen’s Kappa coefficients examined the degree of concordance between the automatic estimates of SA and the Fels reference protocol in classifying participants as early, on-time, and late maturing. All methods presented Kappa coefficient values that were statically significant, thereby indicating agreement between the automatic methods and the reference method (Table 2). The concordance value was highest for the BAUSport system, which presented a good level of agreement (Kappa = .71); and lowest for the BoneXpert TW3 method which demonstrated moderate agreement (Kappa = .35).

**FIG. 3:**
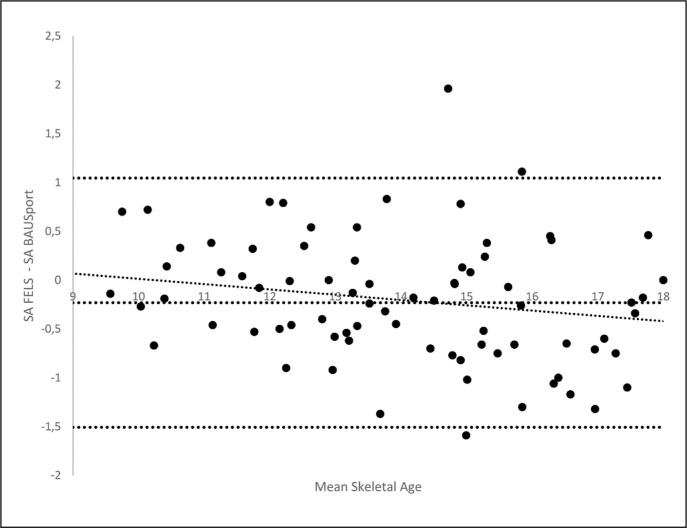
Bland-Atlman plot illustrating the degree of agreement between estimates of skeletal age (SA) derived from the Fels and BAUSport protocols.

Bland-Altman analyses with accompanying linear regression analyses were conducted for each of the automatic estimates of SA and the reference standard. Mean differences (estimated bias) between the estimates of SA and the 95% upper and lower levels of agreement were calculated for each plot (Table 3). A regression line (two-way) was fitted to the scatter plots to identify systematic or proportional biases (Table 3). The estimated mean differences between the automatic estimates of SA and the reference method were all statistically significant (one sample means t-tests), indicating the presence of fixed biases. The estimated biases range from -.23 (BAUSport) to .82 (Bon-eXpert TW3). The range between the 95% upper and lower levels of agreement resulting from the Bland-Altman analyses varied across methods from 1.76 years (BoneXpert Fels) to 2.55 years (BAUSport). None of the methods (presented statically significant associations between the mean estimate of SA and degree of agreement between the estimates of SA. The Bland-Altman plot for the BAUSport estimate of SA and the Fels reference standard is presented in Figure 3.

**TABLE 3:** Bland Altman analyses comparing methods for estimating skeletal age against the Fels method (FELS_PRACT_-Comparison Method) in male and female adolescent athletes aged 11 to 17 years.

	Est. Bias (SD)	ULOA (95%)	LLOA (95%)	LOA Range	r
BAUSport	-.23 (.65)	1.05	-1.50	2.55	-.21
BoneXpert GP	.44 (.46)	1.35	-.46	1.81	-.12
BoneXpert TW3	.82 (.47)	1.74	-.09	1.83	.07
BoneXpert Fels	.22 (.45)	1.10	-.66	1.76	-.07

Note: ULOA = Upper Level of Agreement; LLOA = Lower Level of Agreement.

## DISCUSSION

This study investigated the validity of two automatic methods for estimating skeletal age in athletes aged 9 to 17 years. The ICCs indicated a series of strong and positive associations between the automatic estimates of SA and the Fels reference method. These findings are consistent with previous research using the BAUSport and Bon-eXpert systems [16]. Highly positive ICCs are desirable when comparing estimates of SA in validation studies and suggest a high degree of association between the estimates. They do not, however, reflect the extent to which the estimates of SA agree and/are equivalent to one another. Two methods can be strongly correlated yet produce markedly different estimates of SA. The TW3 method, for example, correlates strongly with other estimates of SA, yet produces lower estimates of SA [11]; as occurred in the current study. Equally, the broad age range of the current sample (9 to 17 years) likely inflated the magnitude of the observed correlations between the estimates of SA. That is, correlations among estimates of SA tend to be smaller when considered in restricted age samples [23]. Thus, these results, although promising, should be interpreted with caution.

The ICCs for the SA-CA discrepancy provided a more rigorous test of validity, as age-associated variance in maturation was controlled for. All the automatic estimates of SA-CA demonstrated positive and statistically significant associations with the reference method. The magnitude of the correlations was strong, varying from .88 (BoneXpert TW3) to.97 (BoneXpert Fels), suggesting that the automatic methods can provide valid estimates of SA-CA discrepancies. This observation is promising as the capacity of sonographic methods to effectively differentiate between children of similar ages, yet varying maturity status, has been questioned [15]. The more fixed geometrical position in which the hand-wrist is positioned when using the BAUSport system and greater number of assessment sites may afford greater validity and reliability when estimating SA via ultrasound.

For the BoneXpert software, the A.TEM and R.TEM values varied from -.35 to -.67 years and 2.60 to 5.07%, respectively, with all three protocols underestimating SA relative to the reference. The A.TEM and R.TEM values were greatest for the BoneXpert TW3 method, which is consistent with previous research [10]. The A.TEM and R.TEM for SA derived via the BAUSport system were comparable to, and fell between, the equivalent values for the BoneXpert estimates. The A.TEM and R.TEM values that are considered acceptable in anthropometry vary relative to the skill of the practitioner, complexity of the assessment, and opportunity for error [24]. Whereas an inter-investigator Relative TEMs of below 7.55% are considered acceptable for less precise measures, such as skinfolds, values below 1.5% are considered acceptable for more precise measures (e.g., height, weight) [24]. As the methods for estimating SA employ separate protocols, one might posit a R.TEM of below 5% to be acceptable in comparing levels of agreement between methods [24]. Applying this criterion, all methods, except for the BoneXpert TW3 protocol, presented R.TEM. values that would be considered acceptable.

The automatic methods for estimating SA all demonstrated statistically significant degrees of agreement with the reference methods in categorizing participants as early, on-time, and late maturing. The non-invasive BAUSport system demonstrated the highest degree of concordance, achieving a good level of agreement, strong enough to be considered clinically significant. The degree of concordance between the BoneXpert and Fels methods varied across protocols, ranging from to moderate (TW3) to good (Fels). Accordingly, both the BAUSport and BoneXpert systems appear to be appropriate methods for identifying youth as early, on time and late maturing.

Although all of methods presented statistically significant fixed biases when compared against the standard; only the BAUSport system presented a negative bias, which is consistent with previous research [16]. None of the methods identified a proportional bias with the Fels refence standard, suggesting no systematic errors associated greater or lesser estimates of SA. The difference between the 95% upper and lower levels of agreement varied across methods, ranging from 1.76 years (BoneXpert Fels) to 2.55 years (BAUSport). The latter finding is worthy of further consideration. Although the BAUS-port system presented the smallest fixed bias and demonstrated the highest level of agreement in categorising participants as early, on-time, and late, it also produced the widest limits of agreement. A closer inspection of the participants that presented the greatest discrepancies between the BAUSport and Fels estimates of SA failed to reveal any influence of participant age and/or maturity status. A potential explanation for the wider levels of agreement is inconsistent use of the BAUSport system. Variance in the positioning of the hand or marking of anatomical sites when using the BAUSport system may have contributed to greater discrepancies in the estimation of SA across cases. More rigorous training on the use of the BAUS-port system and its protocols may be important in terms of determining the degree of training required to optimally ensure methodological fidelity and reduce any extreme errors in estimation of SA.

Practical implications of the current study should be considered. Collectively, the results support the use of the BoneXpert software and BAUSport system as automatic methods for estimating SA in young athletes. The BAUSport system demonstrated the highest level of agreement with the reference method when classifying youth as early, on-time and late maturing. BoneXpert performed best when employing Fels protocol, however, the observation of positive fixed biases across all three protocols indicated a tendency for all three protocols to underestimate SA. Accordingly, estimates of SA derived from the BoneXpert software should be interpreted with caution and not treated as directly interchangeable with values derived from the reference method.

As the BAUSport system does not require exposure to radiation it provides a particular advantage when estimating maturation status in youth; especially in contexts where regular screening and monitoring of growth and maturation status may be advised (e.g., clinical cases, youth sports). In terms of estimating SA and SA-CA the BAUSport method performed as well as the BoneXpert software, although it produced marginally higher estimates of SA than the reference method. Thus, SA estimates derived from the BAUSport system cannot be considered as directly interchangeable with those derived from the reference method. As with all methods, caution is required when interpreting BAUSport estimate of SA at the individual level. The cost-effective, non-invasive, and time-efficient nature of the BAUSport system increases the opportunities for researchers and practitioners performing estimates of SA in countries where specialised equipment or personnel may not be readily available. Ideally all estimates of SA should be considered and interpreted in parallel with other indices of growth and maturation status, such as height/weight velocity, percentage of predicted adult stature, and/or changes in physique, appearance, and/or secondary sex characteristics [10]. The Premier League’s Growth and Maturity Screening Programme, for example, considers multiple sources of information to assess the growth and maturational status of registered academy players every three-to-four months [25]. Combined with non-invasive estimates of SA, such information could provide greater insight as to the physical development of young athletes, optimising their training, athletic development, health, and safety.

Limitations of the current investigation must be noted. First, the results are limited to a small sample of Spanish academy athletes aged 9 to 17 years, the majority of whom were male. It is difficulty to generalise these findings across the sexes or other sports and future studies with larger samples of male and female athletes are required. Male athletes are also more likely to present limited variance in maturity due to inherent selection biases towards early maturers. As maturity selection biases are less common in female sports, female samples may provide more rigorous and representative tests of the validity and reliability of these methods. In contrast, clinical samples tend to demonstrate negative SA-CA discrepancies. The magnitude of the correlations between estimates of SA may also have been artificially inflated relatively broad age range. That said, the strong correlations remained strong for the SA-CA discrepancy, where age associated variance in maturity was effectively controlled for.

## CONCLUSIONS

In conclusion, the current findings support the use of BAUSport as an alternative, practical and non-invasive methods for the estimation of SA in young athletes. In comparison to the established methods for estimating SA in youth, the BAUSport and BoneXpert systems both performed well and especially in relation to the categorization of youth as early, on-time, and delayed in maturation.

## References

[1] Bergeron MF, Mountjoy M, Armstrong N, Chia M, Côté J, Emery CA, et al. International Olympic Committee consensus statement on youth athletic development. Br J Sports Med. 2015; 49(13):843–51. 26084524 10.1136/bjsports-2015-094962

[2] Cumming SP, Lloyd RS, Oliver JL, Eisenmann JC, Malina RM. Bio-banding in sport: Applications to competition, talent identification, and strength and conditioning of youth athletes. Strength Cond J. 2017; 39(2):34–47.

[3] Malina RM, Cumming SP, Rogol AD, Coelho-e-Silva MJ, Figueiredo AJ, Konarski JM, et al. Bio-banding in youth sports: Background, concept, and application. Sports Med. 2019; 49(11)167–1685. 31429034 10.1007/s40279-019-01166-x

[4] Malina RM, Bouchard C, Bar-Or O. Growth, maturation, and physical activity. Human kinetics; 2004.

[5] Greulich W, Pyle, SI. Pyle SI 1959 Radiographic Atlas of Skeletal Development of the Hand and Wrist. Vol. 462. 1959.

[6] Chumela WC, Roche AF, Thissen D. The FELS method of assessing the skeletal maturity of the hand-wrist. Am J Hum Biol. 1989; 1(2):175–83. 28514006 10.1002/ajhb.1310010206

[7] Tanner J, Healy, MJR, Goldstein H, Cameron N. Assessment of skeletal maturity and prediction of adult height (TW3 method). 3rd. Saunders; 2001.

[8] Tanner JM. A new system for estimating skeletal maturity from the hand and wrist, with standards derived from a study of 2600 healthy British children. Part II: The Scoring System. 1959;

[9] Tanner JM. Assessment of skeletal maturity and prediction of adult height. TW 2 Method. 1983; 50–106.

[10] Malina RM. Assessment of biological maturation. In: Oxford textbook of children’s exercise science and medicine. Oxford University Press; 2017. p. 3–11.

[11] Malina RM. Skeletal age and age verification in youth sport. Sports Medicine. 2011; 41(11):925–47. 21985214 10.2165/11590300-000000000-00000

[12] Thodberg HH, Kreiborg S, Juul A, Pedersen KD. The BoneXpert method for automated determination of skeletal maturity. IEEE Trans Med Imag. 2008; 28(1):52–66. 10.1109/TMI.2008.926067 19116188

[13] Mentzel HJ, Vilser C, Eulenstein M, Schwartz T, Vogt S, Böttcher J, et al. Assessment of skeletal age at the wrist in children with a new ultrasound device. Pediatr Radiol. 2005; 35(4):429–33. 15729586 10.1007/s00247-004-1385-3

[14] Castriota-Scanderbeg A, Sacco MC, Emberti-Gialloreti L, Fraracci L. Skeletal age assessment in children and young adults: comparison between a newly developed sonographic method and conventional methods. Skel Radiol. 1998; 27(5):271–7. 10.1007/s002560050380 9638838

[15] Khan KM, Miller BS, Hoggard E, Somani A, Sarafoglou K. Application of ultrasound for bone age estimation in clinical practice. J Pediatr. 2009; 154(2):243–7. 18823906 10.1016/j.jpeds.2008.08.018

[16] Leyhr D, Murr D, Basten L, Eichler K, Hauser T, Lüdin D, et al. Biological maturity status in elite youth soccer players: a comparison of pragmatic diagnostics with magnetic resonance imaging. Front Sports Act Living. 2020; 15; 2:587861 10.3389/fspor.2020.587861 PMC7739788 33345157

[17] Malina RM, Dompier TP, Powell JW, Barron MJ, Moore MT. Validation of a noninvasive maturity estimate relative to skeletal age in youth football players. Clin J of Sport Med. 2007; 17(5):362–8. 17873548 10.1097/JSM.0b013e31815400f4

[18] Faul F, Erdfelder E, Lang AG, Buchner A. G* Power 3: A flexible statistical power analysis program for the social, behavioral, and biomedical sciences. Behav Res Methods. 2007; 39(2):175–91. 17695343 10.3758/bf03193146

[19] Thodberg H, Van Rijn R, Tanaka T, Martin D, Kreiborg S. A paediatric bone index derived by automated radiogrammetry. Osteoporosis Int. 2010; 21(8):1391–400. 10.1007/s00198-009-1085-9 PMC2895878 19937229

[20] Aref Elnasasra M, Hilmi Alnsasra M, Rozalia Smolyakov M, Klaris Riesenberg M, Lior Nesher M. Bone age assessments by quantitative ultrasound (sonicbone) and hand x-ray based methods are comparable. The Israel Medical Association Journal. 2017; September (19), 533–538. 28971634

[21] Rachmiel M, Naugolani L, Mazor-Aronovitch K, Levin A, Koren-Morag N, Bistritzer T. Bone age assessment by a novel quantitative ultrasound based device (SonicBone), is comparable to the conventional Greulich and Pyle method. Horm Res Pediatr. 2013; 80(Suppl 1):35.

[22] Ruf L, Cumming S, Härtel S, Hecksteden A, Drust B, Meyer T. Construct validity of age at predicted adult height and BAUS skeletal age to assess biological maturity in academy soccer. Ann Hum Biol. 2021; 48(2):101–9. 34097548 10.1080/03014460.2021.1913224

[23] Towlson C, MacMaster C, Parr J, Cumming S. One of these things is not like the other: time to differentiate between relative age and biological maturity selection biases in soccer? Sci Med Footb. 2022; 6(3):273–6. 35866421 10.1080/24733938.2021.1946133

[24] Perini TA, Oliveira GL de, Ornellas J dos S, Oliveira FP de. Technical error of measurement in anthropometry. Rev Bras Med Esporte. 2005; 11:81–5.

[25] Cumming SP. A game plan for growth: How football is leading the way in the consideration of biological maturation in young male athletes. Ann Hum Biol. 2018; 45(5):373–5. 30767617 10.1080/03014460.2018.1513560

